# Whole-Genome Sequence Analysis of Antimicrobial Resistance Genes in *Streptococcus uberis* and *Streptococcus dysgalactiae* Isolates from Canadian Dairy Herds

**DOI:** 10.3389/fvets.2017.00063

**Published:** 2017-05-22

**Authors:** Julián Reyes Vélez, Marguerite Cameron, Juan Carlos Rodríguez-Lecompte, Fangfang Xia, Luke C. Heider, Matthew Saab, J. Trenton McClure, Javier Sánchez

**Affiliations:** ^1^Department of Health Management, Atlantic Veterinary College, University of Prince Edward Island, Charlottetown, PE, Canada; ^2^Department of Pathology and Microbiology, Atlantic Veterinary College, University of Prince Edward Island, Charlottetown, PE, Canada; ^3^Mathematics and Computer Science Division, Argonne National Laboratory, Chicago, IL, USA; ^4^Diagnostic Services, Atlantic Veterinary College, University of Prince Edward Island, Charlottetown, PE, Canada

**Keywords:** *Streptococcus uberis*, *Streptococcus dysgalactiae*, antimicrobial resistance, bovine mastitis, whole-genome sequence

## Abstract

The objectives of this study are to determine the occurrence of antimicrobial resistance (AMR) genes using whole-genome sequence (WGS) of *Streptococcus uberis* (*S. uberis*) and *Streptococcus dysgalactiae* (*S. dysgalactiae*) isolates, recovered from dairy cows in the Canadian Maritime Provinces. A secondary objective included the exploration of the association between phenotypic AMR and the genomic characteristics (genome size, guanine–cytosine content, and occurrence of unique gene sequences). Initially, 91 isolates were sequenced, and of these isolates, 89 were assembled. Furthermore, 16 isolates were excluded due to larger than expected genomic sizes (>2.3 bp × 1,000 bp). In the final analysis, 73 were used with complete WGS and minimum inhibitory concentration records, which were part of the previous phenotypic AMR study, representing 18 dairy herds from the Maritime region of Canada (1). A total of 23 unique AMR gene sequences were found in the bacterial genomes, with a mean number of 8.1 (minimum: 5; maximum: 13) per genome. Overall, there were 10 AMR genes [ANT(6), *TEM*-127, *TEM*-163, *TEM*-89, *TEM*-95, *Linb, Lnub, Ermb, Ermc*, and *TetS*] present only in *S. uberis* genomes and 2 genes unique (*EF*-*TU* and *TEM*-71) to the *S. dysgalactiae* genomes; 11 AMR genes [*APH*(3′), *TEM*-1, *TEM*-136, *TEM*-157, *TEM*-47, *TetM, bl2b, gyrA, parE, phoP*, and *rpoB*] were found in both bacterial species. Two-way tabulations showed association between the phenotypic susceptibility to lincosamides and the presence of *linB* (*P* = 0.002) and *lnuB* (*P* < 0.001) genes and the between the presence of *tetM* (*P* = 0.015) and *tetS* (*P* = 0.064) genes and phenotypic resistance to tetracyclines only for the *S. uberis* isolates. The logistic model showed that the odds of resistance (to any of the phenotypically tested antimicrobials) was 4.35 times higher when there were >11 AMR genes present in the genome, compared with <7 AMR genes (*P* < 0.001). The odds of resistance was lower for *S. dysgalactiae* than *S. uberis* (*P* = 0.031). When the within-herd somatic cell count was >250,000 cells/mL, a trend toward higher odds of resistance compared with the baseline category of <150,000 cells/mL was observed. When the isolate corresponded to a post-mastitis sample, there were lower odds of resistance when compared with non-clinical isolates (*P* = 0.01). The results of this study showed the strength of associations between phenotypic AMR resistance of both mastitis pathogens and their genotypic resistome and other epidemiological characteristics.

## Introduction

Bovine mastitis continues to be a priority in the dairy cattle industry, as it is one of the leading causes of reduced profitability, through decreased milk production and increased treatment costs (2). Mastitis is caused by a wide variety of pathogens, with different pathogenic characteristics that damage the mammary gland in a variety of ways (3, 4). Multiple control strategies have been implemented, depending on the epidemiology of the pathogens involved in the intramammary infections (IMIs) (5, 6).

Among all the microbial species causing bovine mastitis, *Streptococcus* spp. are part of a larger group of organisms associated with IMI (7) and have been linked with both clinical and subclinical mastitis (6). *Streptococcus uberis* (*S. uberis*) and *Streptococcus dysgalactiae* (*S. dysgalactiae*) have been classified as Gram-positive, catalase-negative cocci (PNC) microorganisms (8) and are frequently isolated in IMI on Canadian dairy farms (9). *S. uberis* has been reported as having a heterogeneous genotype pattern (6) and associated with environmental transmission sources (10), such as water, soil, plant matter, and bedding material (11). In contrast, *S. dysgalactiae* can spread from infected cows to healthy herd mates (12) or directly from the cow’s environment (13, 14).

Antimicrobial resistance (AMR) has been reported among different mastitis pathogens to various degrees in both short- and long-term studies (15). Phenotypic *in vitro* susceptibility is the ability of an antimicrobial to inhibit the growth of a bacterium in an assay at a concentration above clinical cutoff. However, this indicates high probability of treatment *in vivo*. Recently, patterns of phenotypic antimicrobial *in vitro* susceptibility have been reported for isolates of *S. uberis* and *S. dysgalactiae* (1). These results are our group’s (AMR of the Atlantic Veterinary College) previous work in the area. In that study, isolates of *S. uberis* displayed lower susceptibility and higher minimum inhibitory concentration (MIC) than *S. dysgalactiae* isolates (1). Similarly, in a separate study, multidrug resistance was more common among *S. uberis* isolates than for isolates of *S. dysgalactiae* (16).

The advent of whole-genome sequencing (WGSq) allows the detection of antimicrobial drug resistance determinants and virulence factors genes in mastitis pathogens. Whole-genome sequencing and comparison also allow the investigation of the transmission of bacterial genetic material among host populations with high detail (17, 18). In recent years, there have been several studies using WGS comparisons (the presence of particular sequences) for bovine mastitis pathogens, such as *Escherichia coli* (*E. coli*) (19) and *Staphylococcus aureus* (*S. aureus*) strains (genotyping) (20) and for both *S. uberis* and *S. dysgalactiae* (gene content and comparative genomics) (21, 22). However, there is scarce literature related to the understanding of the AMR genetic potential among bovine mastitis streptococcal species and its relationship with specific epidemiological characteristics such as somatic cell count (SCC) and days in milk (DIM) of the cow.

The primary objectives of this research were to determine the occurrence of unique AMR genes found in *S. uberis* and *S. dysgalactiae* isolates collected from dairy cows in the Maritime Provinces of Canada using WGS analysis and to explore the relationship between phenotypic susceptibility and the presence of AMR resistance genes and epidemiological characteristics. In addition, the secondary objective was to explore the association between phenotypic AMR and the genomic characteristics, such as genome size, guanine–cytosine (GC) content, and number of unique AMR gene sequences, of the isolates.

## Materials and Methods

### Bacterial Collection

*Streptococcus uberis* and *S. dysgalactiae* isolates were obtained from the Mastitis Pathogen Culture Collection of the Canadian Bovine Mastitis and Milk Quality Research Network (CBMQRN) consisting of 16,500 isolates, recovered from 91 commercial dairy herds located in 6 Canadian provinces (23). From this national cohort, 98 isolates included in the Cameron et al.’s (1) study were initially considered for WGS, based on their resistant and pan susceptibility characteristics. Seven isolates were excluded, from the initial number due to low DNA quality. Consequently, 91 isolates of *S. uberis* (*n* = 66) and *S. dysgalactiae* (*n* = 25) were subject to WGS.

### Bacterial Culture

One colony of each isolate was selected and cultured overnight with incubation (6–12 h) at 37°C in 5 mL of Trypticase soy broth (Bacto™ Tryptic Soy Broth, Becton Dickinson, Mississauga, ON, Canada). The grown-up colonies were collected for DNA extraction. A total of 500 µL of each bacterial culture was then removed and added to an equal volume of glycerol freezing solution (65% glycerol, 0.1 M MgSO_4_, 0.025 M Tris–HCl, pH 8.0). Samples were stored at −80°C until needed.

### Determination of Antimicrobial Susceptibility

The antimicrobial susceptibility of each pure subculture was determined by the Sensititre microdilution plate system, containing serial dilutions of eight antimicrobials (ampicillin, ceftiofur, cephalothin, erythromycin, penicillin, penicillin–novobiocin, pirlimycin, and tetracycline). The mastitis plates were read automatically, as described in the study by Cameron et al. (1). The MIC and the susceptibility status were interpreted according to the Clinical Laboratory and Standards Institute guidelines for each of the antimicrobials (CLSI 2013).

### Nucleic Acid Extraction

The DNA extraction was performed using the QIAamp DNA Mini Kit (Qiagen Inc., Canada). A total of 1.5 mL of suspended bacterial culture was pipetted into a 1.5-mL microcentrifuge tube and then pelleted by centrifugation for 10 min at 5,000 × *g*. The bacterial pellet was suspended in 180 µL of lysozyme (20 mg/mL in 20 mM Tris–HCl, pH 8.0; 2 mM EDTA; 1.2% Triton) and incubated at 37°C for 45 min. Then 20 µL of ProtK (Qiagen kit) and 200 µL of buffer AL (Qiagen Kit) were added to each tube and mixed by vortexing. Samples were incubated at 56°C for 1 h. After that, 4 µL of RNase A (100 mg/mL) was added, mixed by vortexing for 15 s, and incubated at 70°C for 60 min. This step was followed by adding 200 µL of ethanol (95–100%) and pulse vortexing for 15 s. Samples were added to the QIAamp mini spin column, and the method was continued according to kit instructions. All samples were eluted with 30 µL of elution buffer from the kit.

### DNA Quantification and DNA Sequencing

From each sample, 2 µL was used to determine the 260:280 ratios for DNA quality and to estimate the DNA concentration using the NanoDrop 1000 spectrophotometer (Thermo Scientific, Wilmington, DE, USA). At least 500 ng of DNA for each sample was added to a 96-well plate and sent for sequencing.

### Whole-Genome Sequencing

Samples for WGS were processed at the Genome British Columbia Genome Sciences Centre. A PCRFree genome library construction from 500 ng of genomic DNA in 62.5 μL of elution buffer (i.e., a concentration of 8 ng/mL) was used. Different qualitative and quantitative control steps were taken for samples at various stages of the library construction (i.e., initial receipt, post-shearing, post-library construction), and these steps were as follows: QCed for quality and quantity with Caliper LabChip GX for DNA samples using the High Sensitivity Assay (PerkinElmer, Inc., USA), Agilent DNA 1000 series II assay, Quant-iT dsDNA HS Assay Kit using Qubit fluorometer (Invitrogen), and KAPA qPCR. A paired-end 125 bp sequencing was performed on the Illumina HiSeq 2500 platform, using V4 chemistry according to manufacturer recommendations. The average coverage was 367×. The overall coverage range was 232×–534×, with the exception of two samples that only got 65×, and therefore, those samples were discarded.

### Bioinformatics and AMR Genes Search

The sequencing center provided libraries in binary alignment files (BAM) against the provided reference genomes for each pathogen species: *S. uberis* 0140J (21) and *S. dysgalactiae* subsp. *dysgalactiae* ATCC 27957 (22). Only Illumina chastity-passed reads were included in the alignment. However, these alignments were not used in this analysis. Instead, the files were transformed back to FASTA format with SAMtools (version 1.3) (24). These genomes were *de novo* assembled using the auto strategy option. This assembly option integrates algorithms for base calling correction (BayesHammer), assembly (Velvet, Spades, IDBA), and scoring (ARAST) giving three separate assemblies and indicating the best assembly (25). Furthermore, the genomes were annotated using the Rast tool kit found in the Pathosystems Resource Integration Center (PATRIC) (PATRIC 3.2.96), as part of the all-bacteria Bioinformatics Resource Center available online (25). The genomes were visualized with the genome browser option, powered by JBrowse (development version), available also in the PATRIC resource (25). Furthermore, these genomes were interrogated for AMR genes, using the specialty genes service of PATRIC 3.2.96 with the AMR filter. This specialty genes filtered data type, including reference sequences of the Comprehensive Antibiotic Resistance Database (CARD) (26) and the Antibiotic Resistance Genes Database (ARDB) (27), which are integrated in the PATRIC tool (http://patricbrc.org). The sequence queries for the specialty genes search were performed with 80% coverage and 80% identity value, including both literature and BLASTP options, described in the PATRIC resource (25).

Other characteristics of the genomes retrieved from the analysis included GC content and genome size. The information regarding AMR genes obtained from the PATRIC tool was downloaded in a tab-delimited format from PATRIC and merged with the phenotypic resistance data used in the study by Cameron et al. (1). This information was processed and analyzed in Stata 13 statistical/data analysis software (28).

### Statistical Analysis

#### Genomic and Antibiotic Resistance Characteristics

Descriptive statistics for the genomic information, such as genome size and GC content, were calculated, stratified by susceptibility phenotype and species (*S. dysgalactiae* and *S. uberis*). Only genomes with sizes <2,300,000 bp (lower than the 75% percentile) were considered for further analysis, since genomes greater than this value could be a result of mixed culture for our population of isolates. The occurrence of identified AMR resistance genes sequences by bacterial species was determined. The number of unique AMR gene sequences per genome was calculated and categorized into three different levels as follows: (1) <7 AMR genes, (2) ≥7–<11 AMR genes, and (3) ≥11 AMR genes. The average within-herd SCC was included as a host factor and categorized as in the study by Cameron et al. (1) into three categories: (1) ≤150,000 cells/mL, (2) 151,000–250,000 cells/mL, and (3) 251,000–400,000 cells/mL. The milk sample type from which the isolate was cultured and placed in one of three categories as follows: clinically healthy lactational milk sample (all isolates recovered in lactation and pre-dry-off sampling) from cows with visually normal milk, post-calving milk sample (collected between 0 and 14 DIM), and post-clinical mastitis milk sample (collected between 2 and 5 weeks after a mastitis event). In addition, the presence of unique AMR genes relevant to each antimicrobial class, among the MIC values, was determined.

Binary variables for each of the three classes of antimicrobials (β-lactam, macrolide, and tetracycline) were constructed based on the presence/absence of AMR gene(s) for that specific class. The occurrence of class-specific AMR genes were compared between phenotypically susceptible versus intermediate/resistant isolates for each of the eight antimicrobials antimicrobial using Fisher’s exact test. Unconditional associations for variable selection between the phenotypic AMR (the outcome variable) and the main epidemiologic variables reported by Cameron et al. (1) were assessed. A statistical significance of *P* ≤ 0.20 was used for the unconditional associations, and further *P* ≤ 0.05 statistical significance criteria were used in the model building process, described below. However, variables with an overall *P* ≤ 0.10 for all categories were retained for informative purposes and shown in the final model. For informative purposes, we left associations in the tables with *P* < 0.10, but we used a *P* < 0.20 for the selection of the variables.

Two separate models were calculated to assess the association between the outcome variable with the number of unique AMR genes sequences (three-level categorical predictor as described above), herd SCCs (three-level categorical predictor as described above), bacterial species (*S. uberis* and *S. dysgalactiae*), and sample type (clinically healthy lactational, post-calving, and post-clinical mastitis milk samples). Isolate and herd were included as random effects. The first model consisted of a two-level logistic model, using isolate and herd as random effects to account for clustering. Moreover, the cow-level clustering was not considered, since a low number of cows contributed to more than one isolate. The second model consisted of a simple logistic regression, to explore the effect of the phenotypic resistance and the same predictor variables, but without the effect of clustering. Interaction was assessed during the model building process, introducing and withdrawing some specific variables to see the change of the magnitude of the coefficients. Furthermore, potential confounding variables were reintroduced and retained if the coefficient varied more than 20%. The model assumptions were examined through residual analysis, and consideration of influential observations and likelihood ratio tests were used to compare both models.

## Results

A total of 89 genomes were assembled and annotated through PATRIC pipelines. The assembly metrics are presented in Table S1 in Supplementary Material for all the genomes, along with the accession numbers in the PATRIC resource. Sixteen isolates were removed from the analysis as genomic size was >2,300,000 bp, suggesting non-pure samples. A total of 73 isolates with complete WGS, representing 18 dairy herds from the Maritime region of Canada, were included. The isolates were distributed among Maritime Provinces as follows: 37% of the isolates were collected in Prince Edward Island (PE), 44% in Nova Scotia, and 19% in New Brunswick. From this population of isolates, 76% were *S. uberis* and 24% were *S. dysgalactiae*.

Table 1 shows the descriptive statistics of the genomic characteristics for both bacterial species stratified by phenotypic resistant status. The overall frequency of phenotypic resistance (intermediate susceptibility or resistance) of the isolates, to one or more of the eight antimicrobials tested was 22%. The proportion of *S. uberis* demonstrating phenotypic AMR was 25%, and the proportion of *S. dysgalactiae* demonstrating phenotypic AMR was 11%.

**Table 1:** **Summary of the genomic characteristics of *Streptococcus uberis* (*n* = 56) and *Streptococcus dysgalactiae* (*n* = 17) isolates by phenotypic antimicrobial susceptibility status**.

Phenotypic status^a^	Statistic	*S. uberis*			*S. dysgalactiae*		
		Genomic size (bp)	% Guanine–cytosine (GC)^b^	Antimicrobial resistance (AMR) genes^c^	Genomic size (bp)	% GC^b^	AMR genes^c^
Resistant	Min	1,860,000	35.5	5	1,960,000	39.2	7
	Median	1,973,000	36.3	10	2,006,000	39.3	9
	Max	2,266,000	36.5	13	2,054,000	39.4	9

Susceptible	Min	1,860,000	35.5	5	1,955,000	39.1	6
	Median	1,960,000	36.3	8	2,006,000	39.3	8
	Max	2,266,000	36.5	13	2,114,000	39.4	9

*Isolates were recovered from dairy cows on 18 herds in the Maritime Provinces of Canada, 2007 and 2008*.

*^a^Antimicrobial susceptibility status for ampicillin, ceftiofur, cephalothin, erythromycin, penicillin, penicillin–novobiocin, pirlimycin, and tetracycline*.

*^b^GC gene content*.

*^c^Number of unique AMR genes per bacterial isolate genome*.

**Figure 1:**
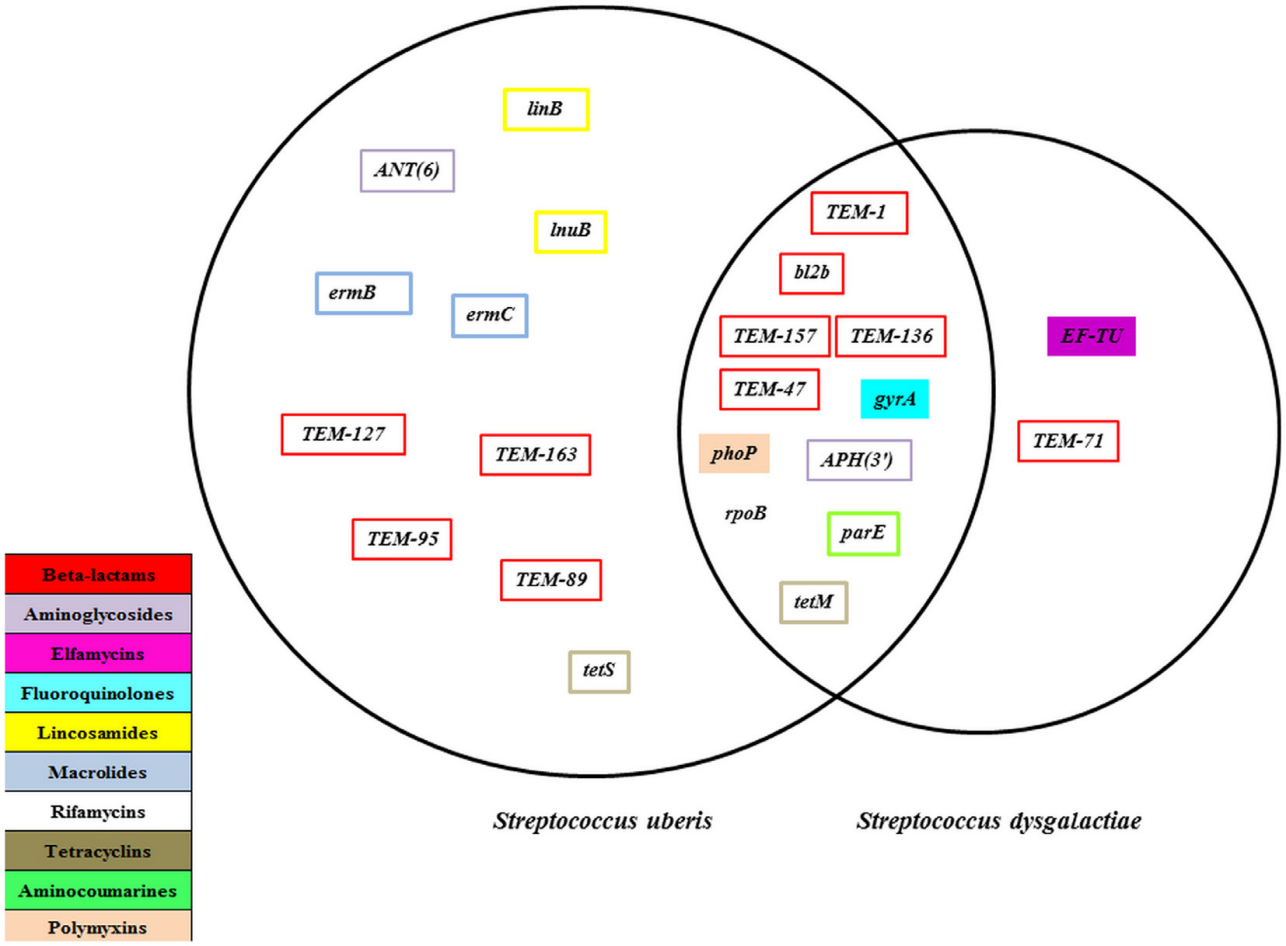
**Antimicrobial resistance genes present for *Streptococcus uberis* (*n* = 64) and *Streptococcus dysgalactiae* (*n* = 25) isolates recovered from dairy herds from the Maritime province of Canada, 2007 and 2008**.

A total of 23 unique AMR genes were found in the bacterial genomes. There were 10 AMR genes sequences found exclusively in *S. uberis* genomes and 2 unique gene sequences in the *S. dysgalactiae* genomes, with the remaining 11 genes shared between both bacterial species (Figure 1). Table 2 shows the occurrence of these genes by bacterial species, the mean identity value, a description of the gene, and the mechanism of resistance. The referred AMR mechanisms were extracted from the Antibiotic Resistance Ontology (26).

**Table 2:** **Occurrence of antimicrobial resistance (AMR) genes and mean identity values identified in the genomes of 56 isolates of *Streptococcus uberis* and 17 isolates of *Streptococcus dysgalactiae* recovered from dairy cows on 18 herds in the Maritime Provinces of Canada, 2007 and 2008**.

Antibiotic group	Grouping gene identifier	Gene	*S. uberis*		*S. dysgalactiae*		Total	Description
			Occurrence^a^	% Identity^b^	Occurrence^b^	% Identity^a^		
Aminocoumarins	*par*	*parE*	56	87.96	17	87.17	73	Topoisomerase IV subunit B

Aminoglycosides	*ant*	*ANT(6)*	33	89.83	–	–	33	Aminoglycoside 6-adenylyltransferase
	*aph*	*APH(3*′*)*	56	99.00	17	99.00	73	

Beta-lactams	*bl2b*	*bl2b*	50	99.00	16	99.00	66	Beta-lactamase
	*tem*	*TEM-1*	4	99.87	2	100.00	6	
		*TEM-127*	1	100.00	–	–	1	
		*TEM-136*	1	100.00	1	100.00	2	
		*TEM-157*	51	99.00	16	99.03	67	
		*TEM-163*	1	99.00	–	–	1	
		*TEM-47*	1	98.00	1	99.00	2	
		*TEM-71*	–		1	99.00	1	
		*TEM-89*	1	100.00	–	–	1	
		*TEM-95*	1	99.00	–	–	1	

Elfamycins	*ef*	*EF-TU*	–	–	1	81.00	1	Translation elongation factor Tu

Fluoroquinolones	*gyr*	*gyrA*	56	80.21	17	80.35	73	DNA gyrase subunit A

Lincosamides	*lin*	*linB*	21	99.00	–	–	21	Putative lincosamide nucleotidyltransferase
	*lnu*	*lnuB*	22	99.97	–	–	22	

Macrolides	*erm*	*ermC*	1	100.00	–	–	1	23S rRNA (adenine(2085)-*N*(6))-dimethyltransferase
		*ermB*	5	99.00	–	–	5	

Polymyxins	*pho*	*phoP*	1	90.00	15	87.00	16	Phosphate regulon transcriptional regulatory protein PhoB

Rifamycins	*rpo*	*rpoB*	56	92.02	16	92.96	72	DNA-directed RNA polymerase beta subunit

Tetracyclines	*tet*	*tetM*	23	99.70	7	–	30	Tetracycline resistance protein TetM
		*tetS*	20	99.50	–	–	20	

*^a^Occurrence: the presence of a unique AMR gene, at least one time in the bacterial genome*.

*^b^% Identity. Two genes with the same residues at the same positions in an alignment, expressed as a percentage*.

The distribution of MIC values for each of the eight antimicrobials by AMR gene and bacterial species is presented in Table S2 in Supplementary Material. *S. uberis* presented higher MICs values than *S. dysgalactiae* for ampicillin, ceftiofur, cephalothin, and penicillin, with some variation when *TEM* genes were present. Also a similar result was observed for pirlimycin and tetracycline, when the respective genes were present. Table S3 in Supplementary Material and Table 3 depict the two-way tabulations between phenotypic susceptibility for each antimicrobial and bacterial species and between the absence/presence of specific AMR genes relevant to each antimicrobial type. Moderate to strong associations were observed in *S. uberis* isolates for ampicillin, ceftiofur, lincosamides, and tetracycline antimicrobials. Moreover, *S. dysgalactiae* showed only moderate associations for cephalothin, penicillin-novobiocin, and tetracycline. The number of total observations for each combination of genes and antimicrobials varied because some specific genomes did not have the presence of specific genes. There were no significant associations between the phenotypic and genotypic variables among the β-lactam antimicrobials.

**Table 3:** **Two-way tabulations used for Fischer’s exact test of the phenotypic susceptibility (resistant/susceptible) against three antimicrobials (pirlimycin, erythromycin, and tetracycline) for isolates of *Streptococcus uberis* (*n* = 56) and *Streptococcus dysgalactiae* (*n* = 17) and the absence/presence of antimicrobial resistance genes categories**.

Antibiotic	*S. uberis*						*S. dysgalactiae*					
	Gene	Genotypic status	Phenotypic status		Total	*P* value	Gene	Genotypic status	Phenotypic status		Total	*P* value
			Resistant	Susceptible					Resistant	Susceptible		
Lincosamides	*linB*	Absent	3	32	35	0.002	*linB*	Absent	1	16	17	N/A
		Present	10	11	21			Present	–	–	–	
	*lnuB*	Absent	2	32	34	<0.001	*lnuB*	Absent	1	16	17	N/A
		Present	11	11	22			Present	–	–	–	

Erythromycin	*ermB*	Absent	10	41	51	1.000	*ermB*	Absent	2	15	17	N/A
		Present	1	4	5			Present				
	*ermC*	Absent	11	44	55	1.000	*ermC*	Absent	2	15	17	N/A
		Present	0	1	1			Present				

Tetracycline	*tetM*	Absent	5	28	33	0.015	*tetM*	Absent	0	10	10	0.154
		Present	11	12	23			Present	2	5	7	
	*tetS*	Absent	7	29	36	0.064	*tetS*	Absent	2	15	17	N/A
		Present	9	11	20			Present				

*Isolates were recovered from dairy cows on 18 farms in the Maritime Provinces of Canada, 2007 and 2008*.

*N/A, there were no observations for this combination of categories*.

The random effects were not significant when accounting for the predictors in the model; therefore, a simple logistic regression model was used. The final simple logistic model was built with 584 observations (8 antibiotics per isolate) and 73 isolates from 17 herds (Table 4). One herd was not included in neither of the two models, because it had only one isolate with complete information. The number of unique AMR gene sequences was significant (*P* < 0.001), and the odds of resistance was 4.34 times greater when there were ≥11 AMR genes present in the genome, compared with genomes with <7 AMR genes. The log odds of resistance was lower for *S. dysgalactiae* than for *S. uberis* (*P* = 0.031). Herds with SCC was >250,000 cells/mL and showed a trend toward higher odds of resistance (*P* = 0.055) compared with the baseline category of <150,000 cells/mL; however, the overall effect of SCC was non-significant (*P* = 0.159). In addition, the sample type categories were significant (*P* = 0.0362). When the isolate was recovered from a post-mastitis sample, the resistance was lower when compared with isolates recovered in non-clinical lactating quarters (*P* = 0.010).

**Table 4:** **Logistic model of AMR against 8 antimicrobials for isolates of *Streptococcus uberis* (*n* = 56) and *Streptococcus dysgalactiae* (*n* = 17) recovered from dairy cows on 17 farms in the Maritime provinces of Canada (2007 and 2008) adjusted by genomic characteristics, antibiotic tested, and AMR gene presence**.

Variable	Odds ratio	SE	*z*	*P*	95% Confidence interval	
					IL	UL
**Number of AMR unique gene sequences**						
**Reference: <7 genes**
≥ 7 < 11	1.871	0.554	2.120	0.034	1.047	3.344
≥11	4.357	1.378	4.650	0.000	2.343	8.100
**Reference: *S. uberis***						
*S. dysgalactiae* SCC	0.495	0.162	−2.150	0.031	0.261	0.939
**Reference: <150,000 cells/mL**						
≥150,00 < 250,000 cells/mL	1.694	0.605	1.480	0.140	0.841	3.412
≥250,000 cells/mL	2.064	0.781	1.920	0.055	0.984	4.331

**Sample type**

**Reference: lactation sample type**
Post-mastitis	0.511	0.134	−2.570	0.010	0.306	0.853
Post-calving	0.732	0.224	−1.020	0.308	0.401	1.334
Intercept	0.132	0.051	−5.190	0.000	0.061	0.283

*AMR, antimicrobial resistance; IL, inferior limit; UL, upper limit; SCC, somatic cell count*.

## Discussion

The combination of phenotypic data (MIC) and WGS information is a novel approach for the field of udder health studies and provides insight into the AMR potential of two major mastitis pathogens in Canadian dairy farms. The occurrence of AMR genes in the whole genomes of *S. uberis* and *S. dysgalactiae* isolates was investigated in relation to phenotypic resistance and with specific epidemiological characteristics. Among both bacterial species, 23 unique AMR genes were detected, distributed differently between *S. uberis* and *S. dysgalactiae* isolates, and conferring potential resistance to different antibiotic groups.

### AMR Genes

The detection of AMR genes obtained through the PATRIC resource (29) was exhaustive since this tool integrates the two main databases of known AMR genes (ARDB and CARD) (26, 27) with standardized genome functional annotation and comparisons pipelines. In general, the average identity value was high for the AMR genes relevant to specific antibiotics used for mastitis treatment: beta-lactams (99.36%), tetracyclines (99.60%), lincosamides (95.15%), and macrolides (96.48%). These high identities could be suggesting that these genes were likely functional. However, the activation of the two-component regulatory system (including the membrane sensor protein and the cytoplasmic response regulator) is necessary to produce gene expression and to trigger inducible genotypes (30). More studies related with the expression of frequently found that genes are needed to further understand the relationship between the phenotype and the genotype of both pathogens.

### Aminocoumarin and Aminoglycosides Resistance Genes

The presence of the *parE, APH(3′)*, and *ANT(6)* genes has been described in other Gram-positive microorganisms such as *S. aureus* (31, 32). The *parE* prevents aminocoumarin antimicrobials from inhibiting DNA synthesis (33). Moreover, *APH*(3′) and *ANT*(6) genes inactivate kanamycin and neomycin and are located in transposons in Gram-positive and Gram-negative bacteria (32, 34, 35). In the current study, *parE* and *APH*(3′) genes were present in both bacterial species with high identity values. In contrast, the *ANT(6)* gene was exclusively present in the *S. uberis* genomes with high identity values. It has been suggested that homologous sequences with more than 40% of identity are likely to share function between them (36). These genes could have been potentially obtained from the dissemination of transposons and plasmids (32) between species cohabiting in the farm environment, especially for *S. uberis* that behaves more as an opportunistic environmental pathogen and is less adapted to the bovine host (11).

### β-Lactams Resistance Genes

One of the most frequent findings in this study was the presence of coding regions for β-lactamase enzymes (*bl2b, TEM-1, TEM-127, TEM-136, TEM-157, TEM-163, TEM-47, TEM-71, TEM-89*, and *TEM-95*). In the present study, as presented in Table 2, the *TEM* variants were more frequent among the *S. uberis* isolates than in the *S. dysgalactiae*. In addition, they have been reported as the most common mechanism of β-lactam resistance in clinically important Gram-negative bacteria (37, 38) such as *Klebsiella pneumoniae* and *E. coli* (39, 40). The presence of encoding regions for β-lactamases in this population of isolates, which are catalase-negative Gram-positive cocci, is a novel finding. However, further molecular confirmation of the presence of these encoding regions and gene expression are needed.

### Elfamycin, Fluoroquinolones, Polymyxin, and Rifamycin Resistance Genes

The presence of elfamycin, fluoroquinolones, polymyxin, and rifamycin resistance genes are presented in Table 2. Elfamycin resistance through the presence of the elongation factor *Tu (EF-TU)* has been described in species of *Staphylococcus, Lactobacillus*, and *Bacillus* (41). However, there is scarce literature regarding elfamycin resistance in streptococci. Also, the *EF-TU* has been used as a genetic marker to perform phylogenetic analysis among streptococcal species (42). The presence of the DNA gyrase (*gyrA*) with mutations have been reported in fluoroquinolone-resistant isolates of *Streptococcus pneumoniae* (43) and *Streptococcus pyogenes* (44). The existence of *gyrA* in both *S. uberis* and *S. dysgalactiae* genomes indicates that it can be expressed as a housekeeping gene regardless the selective pressure. Fluoroquinolones are antimicrobials of high importance in human medicine but rarely used in dairy herds for bovine mastitis treatment (45).

The RNA polymerase beta subunit (*rpoB*) mutations can produce rifamycin resistance in *E. coli, Mycobacterium tuberculosis*, and other microorganisms (46, 47). The high occurrence of this gene in *S. uberis* shown in Table 2 entails a potential public health risk (i.e., rifamycins are used for human tuberculosis).

### Lincosamides, Macrolides, and Tetracyclines Resistance Genes

The resistance to lincosamides through the presence of *linB* and *lnuB* genes has been reported among the streptococci (48–50). In our study, both lincosamide resistance genes were found in the *S. uberis* isolates, but none were identified among the isolates of *S. dysgalactiae* as presented in Table 2. The association between the presence of these two genes and the phenotypic status can suggest potential resistance to other lincosamides, including pirlimycin.

The ribosomal methylase, encoded by the *ermC, ermB*, and *ermR* genes, has been identified as the main determinant of macrolide–lincosamide–streptogramin (MLS) resistance in streptococci, which can be laterally transferred as plasmids between bacteria (48, 51, 52). In the present study, the macrolide–lincosamides resistant genes, such as *ermB* and *ermC*, were present only in the *S. uberis* genomes, similar to the results of Haenni et al. (53), where *S. uberis* isolates carried the genes *linB* and *linD*, implying potential erythromycin and lincomycin phenotypic resistance.

Ribosomal protection proteins genes *tetM* and *tetS* have been described as tetracycline resistance mobile elements in Gram-positive and Gram-negative isolates (53–55). In our study, the *tetM* was present in both bacterial species, and *tetS* was only present in *S. uberis*. The association between the presence of *tetM* and phenotypic status presented in Table 3 in addition to the high-identity mean value (99.5%) could suggest that this gene was expressed within *S. uberis* isolates.

Both streptococci in this study have been classified in the pyogenic group and occupy diverse ecological niches, with pathogenic and commensal characteristics (56). Also, these two species have been classified as low GC Gram-positive bacteria, also known as firmicutes (57). These bacterial genomes can exhibit niche adaptation characteristics, for example, nucleotide usage trends and GC nucleotide distribution (56, 58).

The genome size could potentially influence the phenotypic and genotypic resistance of a pathogen, and it was worth to be explored in the present study. The comparison of genome sizes can be potentially affected by the presence or absence of AMR genes, since those that provide resistance due to point mutations in housekeeping genes could confound this comparison. The genome size has been hypothesized to be associated with the ability of a particular bacterium to develop antibiotic resistance (59). Nonetheless, in the present study, there were no differences in the genome sizes, or GC content, between susceptible and resistant isolates. According to the hypothesis proposed by Projan (59), organisms with larger genomes are more adaptable to environmental changes, and the differences between *S. uberis* and *S. dysgalactiae* isolates can be explained from the proposed “dual nature” (contagious/environmental) of *S. dysgalactiae* (60, 61). In contrast, *S. uberis* has been described as an opportunistic environmental pathogen (11) with lower potential for cow to cow transmission than the *S. dysgalactiae*. The limited survival ability of *S. uberis* in the environment requires constant reintroduction (56). Moreover, the presence of a single strain type in different cows within a herd can be derived from a predominant environmental strain (11).

The GC content is a bacterial taxonomic marker and can vary between 17 and 75% in bacterial species (62). This variation has been attributed to mutation patterns or selective processes and also environmental adaptation (63–65). Moreover, the GC content has been used for distinguishing genes based on their method of transfer, and it is important to characterize AMR genes and pathogenicity islands originated from the exposure to mobile genetic elements (66). The percentage of genomic GC content has been reported to be associated with genome size and the environmental adaptation of the pathogen (67). However, the GC percentage could be influenced by the total GC content originated from successful AMR mobile elements transfers with variable GC content. This might be a potential indicator of the resistome of the microorganism and its probable origin, which needs further investigation.

### Genotypic and Phenotypic Characteristics

Despite the fact that this was mainly a population of susceptible isolates, the presence of β-lactamase enzymes (*TEM-157* and *bl2b*) of plasmid origin (68) was relatively frequent with high MIC values for some specific β-lactams antimicrobials, especially for *S. uberis* isolates. Interestingly, these β-lactamase enzymes were only previously reported in Gram-negative microorganisms (69). A similar scenario was observed for the MLS antimicrobials (lincosamides and macrolides) where the *ermB, linB*, and *lnuB* genes were more frequent among the isolates with higher MIC values. In addition, for both bacterial species, isolates exhibiting *tetM* and *tetS* had higher MICs values. The presence of both genes has been previously reported in bovine and human *Streptococcus* spp. isolates (53, 70). In general, associations between the phenotypic and genotypic characteristics were observed for a limited number of antibiotics for both pathogens (Table 3; Table S3 in Supplementary Material; *P* < 0.10). However, the majority of isolates showed no association between the phenotypic and genotypic characteristics, for some specific antimicrobials. These results can be explained from different approaches, proposed by Gao et al. (71). The first consideration is that gene expression may or may not be present, depending on the existence of a promoter or inducer (71). The second consideration is the possibility that some of the genes need point mutations to be functional and not because of the presence of the gene by itself (71). The third possibility relates to low statistical power in the present study resulting from the small sample size and low number of observations, among combinations of binary predictor variables, representing the phenotypic and genotypic characteristics.

The analysis of genomic variables with phenotypic characteristics is a novel approach to help researchers understand the indicators of host environment adaptation and AMR resistance frequency in dairy herds. The potential resistome is represented here as the number of unique AMR genes and can be related directly with the capacity of developing phenotypic expression; nonetheless, there is scarce literature related to this finding in bovine mastitis pathogens. The expression level of the AMR genes is a key determinant of phenotypic resistance (72), and needs also to be studied, to develop reliable genome-based AMR prediction tools.

In the multivariable model, there was a proportional increase of the log odds of the phenotypic resistance with the number of unique AMR genes. Having more than 11 AMR genes showed increased odds of being phenotypically resistant, when compared to the baseline category. Despite there were variable associations between phenotypic and genotypic characteristics for specific genes and antimicrobials, the overall effect of the number of unique genes showed a significant association with phenotypic resistance. This finding was similar to a recent study, where high agreement between the gene identification in whole genomes of five different bacterial species and their AMR phenotypic characteristics was reported (73). Another study showed high degree of correlation between phenotypic resistance and the presence of one or more relevant resistance genes to a particular antimicrobial in *Campylobacter* species isolates (74). Moreover, the variables such as bacterial species (*S. uberis* compared with *S. dysgalactiae*), different SCC categories (<150,000 cells/mL, ≥150,000 < 250,000 cells/mL, ≥250,000 cells/mL), and sample type (lactation, post-mastitis, and post-calving) had similar effects over the phenotypic resistance as shown in the study by Cameron et al. (1). The isolates from post-mastitis or post-calving milk samples were more likely to be exposed to antimicrobials derived from mastitis treatment and/or from dry period treatments.

## Conclusion

This study showed the distribution of the potential resistome for *S. uberis* and *S. dysgalactiae* isolates through WGS analysis. The presence of specific AMR genes showed a trend toward higher MICs values especially for β-lactams and tetracycline antimicrobials. However, there were no statistically significant associations between the phenotypic and genotypic characteristics for β-lactams and macrolides. On the contrary, there was association between the genotypic and phenotypic characteristics, relevant to lincosamides and tetracycline antimicrobials. The results of this research showed the association between phenotypic resistance and the number of unique AMR gene sequences. There were differences in the phenotypic and genotypic resistance between bacterial species. Interestingly, these results showed that subclinical isolates can harbor AMR genes as well and should be considered as potential propagators of the AMR resistance in dairy herds. Some specific AMR genes found in these genomes could be potentially originated from horizontal transfer of mobile elements. In general, more studies are needed to explore the potential sources of these AMR genes, especially in this pyogenic group of pathogens.

## Author Contributions

JV was the primary author of this manuscript and was responsible for the bioinformatics and data analysis with the participation of MC, FX, and JS. The rest of the authors were involved in the design, analysis, and interpretation of these results. All the authors reviewed critically the manuscript.

## Conflict of Interest Statement

None of the authors of this manuscript have financial or personal relationship with organizations or other people that could influence the content of this study and represent potential conflict of interest. The reviewer, GM, and handling editor declared their shared affiliation, and the handling editor states that the process nevertheless met the standards of a fair and objective review.
