# Supplementary material for: Whole-Genome Sequence Analysis of Antimicrobial Resistance Genes in Streptococcus uberis and Streptococcus dysgalactiae Isolates from Canadian Dairy Herds
Source: Front Vet Sci. 2017 May 22;4:63. doi: 10.3389/fvets.2017.00063 (PMC5438997; doi:10.3389/fvets.2017.00063)
Supplement: Supplementary file 1 [file data_sheet_1.docx]

***Supplementary Material***

**Whole-genome sequence analysis of antimicrobial resistance genes in *Streptococcus uberis* and *Streptococcus dysgalactiae* isolates from Canadian dairy herds.**

Julián Reyes ^*^, Marguerite Cameron, Juan Carlos Rodríguez-Lecompte, Fang Fang Xia, Luke Heider, Matthew Saab, J. Trenton McClure and Javier Sánchez.

*** Correspondence:** Corresponding Author: **jreyes@upei.ca.**

**Supplementary Tables**

Table S1. Whole genome sequence Accession Numbers and assembly metrics for *Streptococcus uberis* (n=64) and *Streptococcus dysgalactiae* (n=25) isolates recovered from dairy herds from the Maritime province of Canada, 2007 and 2008.

| Genome Accession Number | Contigs | Genome Length | GC Content^2^ | PATRIC CDS^3^ |
| --- | --- | --- | --- | --- |
| 1334.34 | 73 | 2,005,178 | 39.3 | 2044 |
| 1334.35 | 69 | 2,023,126 | 39.31 | 2080 |
| 1334.36 | 94 | 2,011,522 | 39.3 | 2069 |
| 1334.37 | 70 | 2,010,978 | 39.29 | 2066 |
| 1334.39 | 113 | 2,113,617 | 39.18 | 2189 |
| 1334.40 | 74 | 2,005,905 | 39.3 | 2035 |
| 1334.41 | 71 | 2,004,671 | 39.21 | 2036 |
| 1334.42 | 74 | 1,955,080 | 39.33 | 1994 |
| 1334.43 | 71 | 2,005,784 | 39.29 | 2035 |
| 1334.44 | 76 | 1,993,774 | 39.45 | 2015 |
| 1334.45 | 235 | 4,296,880 ^1^ | 37.33 | 4389 |
| 1334.46 | 325 | 4,599,768^1^ | 37.4 | 4895 |
| 1334.48 | 161 | 3,885,709^1^ | 37.89 | 3944 |
| 1334.50 | 78 | 2,054,427 | 39.42 | 2094 |
| 1334.51 | 75 | 2,010,737 | 39.29 | 2064 |
| 1334.52 | 89 | 2,004,922 | 39.42 | 2022 |
| 1334.53 | 670 | 4,656,157^1^ | 37.24 | 5158 |
| 1334.54 | 193 | 4,146,618^1^ | 37.56 | 4220 |
| 1334.55 | 445 | 4,176,678^1^ | 37.66 | 4487 |
| 1334.56 | 523 | 4,416,824^1^ | 37.21 | 4815 |
| 1334.57 | 77 | 1,960,348 | 39.31 | 1999 |
| 1334.58 | 93 | 2,069,842 | 39.24 | 2134 |
| 1334.88 | 344 | 4,299,855^1^ | 37.71 | 4575 |
| 1334.97 | 105 | 2,241,600 | 39.03 | 2347 |
| 1334.99 | 54 | 1,960,806 | 39.39 | 1997 |
| 1349.101 | 26 | 1,963,224 | 36.34 | 1973 |
| 1349.111 | 314 | 2,607,672^1^ | 35.35 | 2789 |
| 1349.115 | 20 | 1,982,596 | 36.28 | 1971 |
| 1349.116 | 11 | 1,934,723 | 36.36 | 1933 |
| 1349.117 | 284 | 2,411,852^1^ | 35.41 | 2563 |
| 1349.134 | 14 | 1,920,408 | 36.48 | 1900 |
| 1349.135 | 34 | 1,954,756 | 36.26 | 1982 |
| 1349.136 | 16 | 1,881,126 | 36.44 | 1868 |
| 1349.138 | 70 | 2,151,766 | 36.14 | 2151 |
| 1349.139 | 130 | 2,191,923 | 36.08 | 2266 |
| 1349.140 | 228 | 2,250,548 | 35.77 | 2348 |
| 1349.145 | 16 | 1,935,611 | 36.42 | 1912 |
| 1349.148 | 10 | 1,882,003 | 36.45 | 1899 |
| 1349.185 | 24 | 1,921,399 | 36.39 | 1937 |
| 1349.196 | 14 | 1,880,129 | 36.43 | 1864 |
| 1349.197 | 18 | 1,866,363 | 36.42 | 1840 |
| 1349.198 | 20 | 1,995,756 | 36.23 | 1980 |
| 1349.199 | 14 | 1,920,844 | 36.47 | 1918 |
| 1349.200 | 14 | 1,994,520 | 36.2 | 1975 |
| 1349.201 | 440 | 2,542,813^1^ | 35.16 | 2823 |
| 1349.202 | 23 | 2,095,269 | 36.48 | 2132 |
| 1349.203 | 15 | 1,951,160 | 36.26 | 1970 |
| 1349.204 | 13 | 1,880,761 | 36.43 | 1865 |
| 1349.205 | 14 | 1,941,582 | 36.29 | 1956 |
| 1349.206 | 33 | 1,985,330 | 36.45 | 2037 |
| 1349.207 | 21 | 1,950,075 | 36.37 | 1964 |
| 1349.208 | 221 | 2,196,595 | 35.61 | 2353 |
| 1349.209 | 14 | 1,868,393 | 36.4 | 1878 |
| 1349.210 | 231 | 2,266,934 | 35.57 | 2423 |
| 1349.211 | 10 | 1,941,579 | 36.43 | 1948 |
| 1349.212 | 21 | 2,092,967 | 36.48 | 2128 |
| 1349.213 | 17 | 1,973,325 | 36.34 | 2000 |
| 1349.214 | 26 | 2,017,272 | 36.22 | 2012 |
| 1349.215 | 26 | 1,924,856 | 36.39 | 1935 |
| 1349.216 | 192 | 2,212,252 | 35.87 | 2321 |
| 1349.217 | 169 | 2,254,620 | 36.03 | 2390 |
| 1349.218 | 64 | 2,100,818 | 36.29 | 2133 |
| 1349.219 | 15 | 1,922,316 | 36.31 | 1919 |
| 1349.220 | 22 | 1,945,627 | 36.33 | 1929 |
| 1349.221 | 12 | 1,862,907 | 36.41 | 1867 |
| 1349.222 | 11 | 1,860,725 | 36.41 | 1865 |
| 1349.223 | 14 | 1,918,203 | 36.28 | 1900 |
| 1349.224 | 94 | 2,210,742 | 35.88 | 2256 |
| 1349.225 | 365 | 2,433,319^1^ | 35.49 | 2693 |
| 1349.226 | 20 | 2,063,947 | 36.19 | 2084 |
| 1349.227 | 59 | 2,160,332 | 36.04 | 2201 |
| 1349.228 | 26 | 2,093,654 | 36.48 | 2130 |
| 1349.229 | 16 | 1,959,470 | 36.39 | 1966 |
| 1349.230 | 15 | 1,961,456 | 36.51 | 1979 |
| 1349.231 | 22 | 1,942,362 | 36.38 | 1928 |
| 1349.232 | 13 | 1,993,025 | 36.15 | 1960 |
| 1349.233 | 334 | 2,739,608^1^ | 35.27 | 2993 |
| 1349.30 | 22 | 1,974,975 | 36.32 | 1996 |
| 1349.32 | 191 | 2,327,099^1^ | 35.67 | 2500 |
| 1349.33 | 53 | 2,153,430 | 36.21 | 2193 |
| 1349.35 | 184 | 2,300,283^1^ | 36.08 | 2429 |
| 1349.39 | 75 | 2,047,572 | 36.38 | 2080 |
| 1349.42 | 24 | 1,946,126 | 36.33 | 1927 |
| 1349.47 | 29 | 2,003,632 | 36.4 | 2038 |
| 1349.57 | 15 | 1,915,345 | 36.44 | 1908 |
| 1349.61 | 16 | 1,968,232 | 36.35 | 1971 |
| 1349.73 | 14 | 1,892,622 | 36.35 | 1872 |
| 1349.75 | 19 | 2,012,461 | 36.27 | 2092 |
| 1349.84 | 689 | 4,679,663^1^ | 37 | 5204 |

^1^ Genomes excluded from the analysis

^2^ Guanine cytosine content

^3^ Coding DNA sequence

Table S2.Distribution of minimum inhibitory concentrations (MIC) against eight antimicrobials by AMR genes identified in the genomes of *Streptococcus uberis* (n= 56) *Streptococcus dysgalactiae* (n=17) isolates recovered from dairy cows on 18 herds in the Maritime Provinces of Canada, 2007 and 2008.

| Antibiotic | Species | GENES | Distribution of MIC (µg/mL) | | | | | | | | | Total |
| --- | --- | --- | --- | --- | --- | --- | --- | --- | --- | --- | --- | --- |
|  |  |  | 0.0625 | 0.125 | 0.25 | 0.5 | 1 | 2 | 4 | 8 | 16 |  |
| Ampicillin | *S. uberis* | *TEM-1* |  | 1 | 2 | 1 |  |  |  |  |  | 4 |
|  |  | *TEM-47* |  |  | 1 |  |  |  |  |  |  | 1 |
|  |  | *TEM-71* |  |  |  |  |  |  |  |  |  |  |
|  |  | *TEM-89* |  |  |  | 1 |  |  |  |  |  | 1 |
|  |  | *TEM-95* |  |  | 1 |  |  |  |  |  |  | 1 |
|  |  | *TEM-127* |  |  |  | 1 |  |  |  |  |  | 1 |
|  |  | *TEM-136* |  |  |  | 1 |  |  |  |  |  | 1 |
|  |  | *TEM-157* | 6 | 2 | 25 | 14 | 1 | 1 | 2 |  |  | 51 |
|  |  | *TEM-163* |  |  |  | 1 |  |  |  |  |  | 1 |
|  |  | *bl2b* | 6 | 3 | 23 | 15 | 1 | 2 |  |  |  | 50 |
|  | *S. dysgalactiae* | *TEM-1* | 2 |  |  |  |  |  |  |  |  | 2 |
|  |  | *TEM-47* |  |  | 1 |  |  |  |  |  |  | 1 |
|  |  | *TEM-71* | 1 |  |  |  |  |  |  |  |  | 1 |
|  |  | *TEM-89* |  |  |  |  |  |  |  |  |  |  |
|  |  | *TEM-95* |  |  |  |  |  |  |  |  |  |  |
|  |  | *TEM-127* |  |  |  |  |  |  |  |  |  |  |
|  |  | *TEM-136* | 1 |  |  |  |  |  |  |  |  | 1 |
|  |  | *TEM-157* | 15 |  | 1 |  |  |  |  |  |  | 16 |
|  |  | *TEM-163* |  |  |  |  |  |  |  |  |  |  |
|  |  | *bl2b* | 15 |  | 1 |  |  |  |  |  |  | 16 |
| Ceftiofur | *S. uberis* | *TEM-1* |  |  |  | 2 | 2 |  |  |  |  | 4 |
|  |  | *TEM-47* |  |  |  | 1 |  |  |  |  |  | 1 |
|  |  | *TEM-71* |  |  |  |  |  |  |  |  |  |  |
|  |  | *TEM-89* |  |  |  |  |  | 1 |  |  |  | 1 |
|  |  | *TEM-95* |  |  |  |  | 1 |  |  |  |  | 1 |
|  |  | *TEM-127* |  |  |  |  | 1 |  |  |  |  | 1 |
|  |  | *TEM-136* |  |  |  |  | 1 |  |  |  |  | 1 |
|  |  | *TEM-157* |  |  | 10 | 9 | 26 | 6 |  |  |  | 51 |
|  |  | *TEM-163* |  |  |  | 1 |  |  |  |  |  | 1 |
|  |  | *bl2b* |  |  | 9 | 8 | 27 | 6 |  |  |  | 50 |
|  | *S. dysgalactiae* | *TEM-1* |  |  | 2 |  |  |  |  |  |  | 2 |
|  |  | *TEM-47* |  |  | 1 |  |  |  |  |  |  | 1 |
|  |  | *TEM-71* |  |  | 1 |  |  |  |  |  |  | 1 |
|  |  | *TEM-89* |  |  |  |  |  |  |  |  |  |  |
|  |  | *TEM-95* |  |  |  |  |  |  |  |  |  |  |
|  |  | *TEM-127* |  |  |  |  |  |  |  |  |  |  |
|  |  | *TEM-136* |  |  | 1 |  |  |  |  |  |  | 1 |
|  |  | *TEM-157* |  |  | 15 | 1 |  |  |  |  |  | 16 |
|  |  | *TEM-163* |  |  |  |  |  |  |  |  |  |  |
|  |  | *bl2b* |  |  | 15 |  | 1 |  |  |  |  | 16 |
| Cephalothin | *S. uberis* | *TEM-1* |  |  |  |  | 4 |  |  |  |  | 4 |
|  |  | *TEM-47* |  |  |  |  | 1 |  |  |  |  | 1 |
|  |  | *TEM-71* |  |  |  |  |  |  |  |  |  |  |
|  |  | *TEM-89* |  |  |  |  |  | 1 |  |  |  | 1 |
|  |  | *TEM-95* |  |  |  |  | 1 |  |  |  |  | 1 |
|  |  | *TEM-127* |  |  |  |  |  | 1 |  |  |  | 1 |
|  |  | *TEM-136* |  |  |  |  | 1 |  |  |  |  | 1 |
|  |  | *TEM-157* |  |  |  |  | 46 | 6 | 1 | 1 |  | 51 |
|  |  | *TEM-163* |  |  |  |  | 1 |  |  |  |  | 1 |
|  |  | *bl2b* |  |  |  |  | 42 | 6 |  | 1 | 1 | 50 |
|  | *S. dysgalactiae* | *TEM-1* |  |  |  |  | 2 |  |  |  |  | 2 |
|  |  | *TEM-47* |  |  |  |  | 1 |  |  |  |  | 1 |
|  |  | *TEM-71* |  |  |  |  | 1 |  |  |  |  | 1 |
|  |  | *TEM-89* |  |  |  |  |  |  |  |  |  |  |
|  |  | *TEM-95* |  |  |  |  |  |  |  |  |  |  |
|  |  | *TEM-127* |  |  |  |  |  |  |  |  |  |  |
|  |  | *TEM-136* |  |  |  |  | 1 |  |  |  |  | 1 |
|  |  | *TEM-157* |  |  |  |  | 15 |  | 1 |  |  | 16 |
|  |  | *TEM-163* |  |  |  |  |  |  |  |  |  |  |
|  |  | *bl2b* |  |  |  |  | 15 |  | 1 |  |  | 16 |
| Erythromycin * | *S.uberis* | *ermB* |  |  |  |  |  |  | 5 |  |  | 5 |
|  |  | *ermC* |  |  |  |  |  |  | 1 |  |  | 1 |
| Penicillin | *S. uberis* | *TEM-1* |  | 1 | 1 | 2 |  |  |  |  |  | 4 |
|  |  | *TEM-47* |  |  |  | 1 |  |  |  |  |  | 1 |
|  |  | *TEM-71* |  |  |  |  |  |  |  |  |  |  |
|  |  | *TEM-89* |  |  |  | 1 |  |  |  |  |  | 1 |
|  |  | *TEM-95* |  |  | 1 |  |  |  |  |  |  | 1 |
|  |  | *TEM-127* |  |  |  | 1 |  |  |  |  |  | 1 |
|  |  | *TEM-136* |  |  |  | 1 |  |  |  |  |  | 1 |
|  |  | *TEM-157* | 6 | 4 | 21 | 14 | 3 |  | 3 |  |  | 51 |
|  |  | *TEM-163* |  |  |  |  |  |  |  |  |  |  |
|  |  | *bl2b* | 6 | 4 | 19 | 16 | 2 |  | 3 |  |  | 50 |
|  | *S. dysgalactiae* | *TEM-1* | 2 |  |  |  |  |  |  |  |  | 2 |
|  |  | *TEM-47* | 1 |  |  |  |  |  |  |  |  | 1 |
|  |  | *TEM-71* | 1 |  |  |  |  |  |  |  |  | 1 |
|  |  | *TEM-89* |  |  |  |  |  |  |  |  |  |  |
|  |  | *TEM-95* |  |  |  |  |  |  |  |  |  |  |
|  |  | *TEM-127* |  |  |  |  |  |  |  |  |  |  |
|  |  | *TEM-136* | 1 |  |  |  |  |  |  |  |  | 1 |
|  |  | *TEM-157* | 14 | 1 |  | 1 |  |  |  |  |  | 16 |
|  |  | *TEM-163* |  |  |  | 1 |  |  |  |  |  | 1 |
|  |  | *bl2b* | 14 | 1 |  | 1 |  |  |  |  |  | 16 |
| Penicillin/ novobiocin | *S. uberis* | *TEM-1* |  |  |  | 4 |  |  |  |  |  | 4 |
|  |  | *TEM-47* |  |  |  | 1 |  |  |  |  |  | 1 |
|  |  | *TEM-71* |  |  |  |  |  |  |  |  |  |  |
|  |  | *TEM-89* |  |  |  | 1 |  |  |  |  |  | 1 |
|  |  | *TEM-95* |  |  |  |  | 1 |  |  |  |  | 1 |
|  |  | *TEM-127* |  |  |  | 1 |  |  |  |  |  | 1 |
|  |  | *TEM-136* |  |  |  | 1 |  |  |  |  |  | 1 |
|  |  | *TEM-157* |  |  |  | 46 | 5 |  |  |  |  | 51 |
|  |  | *TEM-163* |  |  |  | 1 |  |  |  |  |  | 1 |
|  |  | *bl2b* |  |  |  | 46 | 4 |  |  |  |  | 50 |
|  | *S. dysgalactiae* | *TEM-1* |  |  |  | 2 |  |  |  |  |  | 2 |
|  |  | *TEM-47* |  |  |  | 1 |  |  |  |  |  | 1 |
|  |  | *TEM-71* |  |  |  | 1 |  |  |  |  |  | 1 |
|  |  | *TEM-89* |  |  |  |  |  |  |  |  |  |  |
|  |  | *TEM-95* |  |  |  |  |  |  |  |  |  |  |
|  |  | *TEM-127* |  |  |  |  |  |  |  |  |  |  |
|  |  | *TEM-136* |  |  |  | 1 |  |  |  |  |  | 1 |
|  |  | *TEM-157* |  |  |  | 15 | 1 |  |  |  |  | 16 |
|  |  | *TEM-163* |  |  |  |  |  |  |  |  |  |  |
|  |  | *bl2b* |  |  |  | 15 | 1 |  |  |  |  | 16 |
| Pirlimycin* | *S. uberis* | *linB* |  |  | 1 |  |  | 7 | 13 |  |  | 21 |
|  |  | *lnuB* |  |  | 1 |  |  | 7 | 14 |  |  | 22 |
| Tetracycline | *S. uberis* | *tetM* |  |  |  |  |  |  |  | 23 |  | 23 |
|  |  | *tetS* |  |  |  |  | 20 |  |  |  |  | 20 |
|  | *S. dysgalactiae* | *tetM* |  |  |  |  |  |  |  | 7 |  | 7 |
|  |  | *tetS* |  |  |  |  |  |  |  |  |  |  |

^1^ Antimicrobial resistance genes

^2^ Solid grey indicate CLSI (2013; 2014) breakpoints

Table S3. Two-way tabulations used for Fischer’s exact test of the phenotypic susceptibility (resistant/susceptible) against 5 β-lactam antimicrobials for isolates of *Streptococcus uberis* (n =56) and *Streptococcus dysgalactiae* (n =17) and the absence/presence of antimicrobial resistance genes categories. Isolates were recovered from dairy cows on 18 farms in the Maritime Provinces of Canada, 2007 and 2008.

| Antibiotic | Genes | Genotypic status | *Streptococcus uberis* | | | *Streptococcus dysgalactiae* | | |
| --- | --- | --- | --- | --- | --- | --- | --- | --- |
|  |  |  | % Resistant | Total | *P* value | % Resistant | Total | *P* value |
|  |  |  |  |  |  |  |  |  |
| Ampicillin | *TEM-1* | *Absent* | 28.8 | 52 | * | 26.7 | 15 | * |
|  |  | *Present* | 25.0 | 4 |  | 50.0 | 2 |  |
|  | *TEM-47* | *Absent* | 29.1 | 55 | * | 25.0 | 16 | * |
|  |  | *Present* | 0.0 | 1 |  | 100.0 | 1 |  |
|  | *TEM-71* | *Absent* | 28.6 | 56 | N/A | 31.3 | 16 | * |
|  |  | *Present* |  |  |  | 0.0 | 1 |  |
|  | *TEM-89* | *Absent* | 27.3 | 55 | ** | 29.4 | 17 | N/A |
|  |  | *Present* | 100.0 | 1 |  |  |  |  |
|  | *TEM-95* | *Absent* | 27.3 | 55 | ** | 29.4 | 17 | N/A |
|  |  | *Present* | 100.0 | 1 |  |  |  |  |
|  | *TEM-127* | *Absent* | 27.3 | 55 | ** | 29.4 | 17 | N/A |
|  |  | *Present* | 100.0 | 1 |  |  |  |  |
|  | *TEM-136* | *Absent* | 29.1 | 55 | * | 31.3 | 16 | * |
|  |  | *Present* | 0.0 | 1 |  | 0.0 | 1 |  |
|  | *TEM-157* | *Absent* | 40.0 | 5 | * | 0.0 | 1 | * |
|  |  | *Present* | 27.5 | 51 |  | 31.3 | 16 |  |
|  | *TEM-163* | *Absent* | 100.0 | 16 | * | 29.4 | 17 | N/A |
|  |  | *Present* | 97.5 | 40 |  |  |  |  |
|  | *BL2B* | *Absent* | 33.3 | 6 | * | 100.0 | 1 | * |
|  |  | *Present* | 28.0 | 50 |  | 25.0 | 16 |  |
| Ceftiofur | *TEM-1* | *Absent* | 32.7 | 52 | ** | 6.7 | 15 | * |
|  |  | *Present* | 75.0 | 4 |  | 0.0 | 2 |  |
|  | *TEM-47* | *Absent* | 34.5 | 55 | * | 6.3 | 16 | * |
|  |  | *Present* | 100.0 | 1 |  | 0.0 | 1 |  |
|  | *TEM-71* | *Absent* | 35.7 | 56 | N/A | 6.3 | 16 | * |
|  |  | *Present* |  |  |  | 0.0 | 1 |  |
|  | *TEM-89* | *Absent* | 36.4 | 55 | * | 5.9 | 17 | N/A |
|  |  | *Present* | 0.0 | 1 |  |  |  |  |
|  | *TEM-95* | *Absent* | 34.5 | 55 | * | 5.9 | 17 | N/A |
|  |  | *Present* | 100.0 | 1 |  |  |  |  |
|  | *TEM-127* | *Absent* | 34.5 | 55 | * | 5.9 | 17 | N/A |
|  |  | *Present* | 100.0 | 1 |  |  |  |  |
|  | *TEM-136* | *Absent* | 36.4 | 55 | * | 6.3 | 16 | * |
|  |  | *Present* | 0.0 | 1 |  | 0.0 | 1 |  |
|  | *TEM-157* | *Absent* | 60.0 | 5 | * | 0.0 | 1 | * |
|  |  | *Present* | 33.3 | 51 |  | 6.3 | 16 |  |
|  | *TEM-163* | *Absent* | 36.4 | 55 | * | 5.9 | 17 | * |
|  |  | *Present* | 0.0 | 1 |  |  |  |  |
|  | *BL2B* | *Absent* | 50.0 | 6 | * | 0.0 | 1 | * |
|  |  | *Present* | 34.0 | 50 |  | 6.3 | 16 |  |
| Cephalotin | *TEM-1* | *Absent* | 23.1 | 52 | * | 0.0 | 15 | ** |
|  |  | *Present* | 0.0 | 4 |  | 50.0 | 2 |  |
|  | *TEM-47* | *Absent* | 21.8 | 55 | * | 6.3 | 16 | * |
|  |  | *Present* | 0.0 | 1 |  | 0.0 | 1 |  |
|  | *TEM-71* | *Absent* | 21.4 | 56 | N/A | 0.0 | 16 | *** |
|  |  | *Present* |  |  |  | 100.0 | 1 |  |
|  | *TEM-89* | *Absent* | 21.8 | 55 | * | 5.9 | 17 | N/A |
|  |  | *Present* | 0.0 | 1 |  |  |  |  |
|  | *TEM-95* | *Absent* | 21.8 | 55 | * | 5.9 | 17 | N/A |
|  |  | *Present* | 0.0 | 1 |  |  |  |  |
|  | *TEM-127* | *Absent* | 21.8 | 55 | * | 5.9 | 17 | N/A |
|  |  | *Present* | 0.0 | 1 |  |  |  |  |
|  | *TEM-136* | *Absent* | 20.0 | 55 | * | 6.3 | 16 | * |
|  |  | *Present* | 100.0 | 1 |  | 0.0 | 1 |  |
|  | *TEM-157* | *Absent* | 0.0 | 5 | * | 100.0 | 1 | *** |
|  |  | *Present* | 23.5 | 51 |  | 0.0 | 16 |  |
|  | *TEM-163* | *Absent* | 21.8 | 55 | * | 5.9 | 17 | N/A |
|  |  | *Present* | 0.0 | 1 |  |  |  |  |
|  | *bl2b* | *Absent* | 16.7 | 6 | * | 0.0 | 1 | * |
|  |  | *Present* | 22.0 | 50 |  | 6.3 | 16 |  |
| Penicillin | *TEM-1* | *Absent* | 28.8 | 52 | * | 28.8 | 52 | * |
|  |  | *Present* | 0.0 | 4 |  | 0.0 | 4 |  |
|  | *TEM-47* | *Absent* | 27.3 | 55 | * | 27.3 | 55 | * |
|  |  | *Present* | 0.0 | 1 |  | 0.0 | 1 |  |
|  | *TEM-71* | *Absent* | 26.8 | 56 | N/A | 26.8 | 56 | N/A |
|  |  | *Present* |  |  |  |  |  |  |
|  | *TEM-89* | *Absent* | 27.3 | 55 | * | 27.3 | 55 | * |
|  |  | *Present* | 0.0 | 1 |  | 0.0 | 1 |  |
|  | *TEM-95* | *Absent* | 27.3 | 55 | * | 27.3 | 55 | * |
|  |  | *Present* | 0.0 | 1 |  | 0.0 | 1 |  |
|  | *TEM-127* | *Absent* | 25.5 | 55 | * | 25.5 | 55 | * |
|  |  | *Present* | 100.0 | 1 |  | 100.0 | 1 |  |
|  | *TEM-136* | *Absent* | 25.5 | 55 | * | 25.5 | 55 | * |
|  |  | *Present* | 100.0 | 1 |  | 100.0 | 1 |  |
|  | *TEM-157* | *Absent* | 0.0 | 5 | * | 0.0 | 5 | * |
|  |  | *Present* | 29.4 | 51 |  | 29.4 | 51 |  |
|  | *TEM-163* | *Absent* | 27.3 | 55 | * | 27.3 | 55 | * |
|  |  | *Present* | 0.0 | 1 |  | 0.0 | 1 |  |
|  | *bl2b* | *Absent* | 33.3 | 6 | * | 33.3 | 6 | * |
|  |  | *Present* | 26.0 | 50 |  | 26.0 | 50 |  |
| Penicillin-novobiocin | *TEM-1* | *Absent* | 19.2 | 52 | * | 19.2 | 52 | * |
|  |  | *Present* | 0.0 | 56 |  | 0.0 | 4 |  |
|  | *TEM-47* | *Absent* | 18.2 | 55 | * | 18.2 | 55 | * |
|  |  | *Present* | 0.0 | 1 |  | 0.0 | 1 |  |
|  | *TEM-71* | *Absent* | 17.9 | 56 | N/A | 17.9 | 56 | N/A |
|  |  | *Present* |  |  |  |  |  |  |
|  | *TEM-89* | *Absent* | 16.4 | 55 | * | 16.4 | 55 | ** |
|  |  | *Present* | 100.0 | 1 |  | 100.0 | 1 |  |
|  | *TEM-95* | *Absent* | 18.2 | 55 | * | 18.2 | 55 | * |
|  |  | *Present* | 0.0 | 1 |  | 0.0 | 1 |  |
|  | *TEM-127* | *Absent* | 18.2 | 55 | * | 18.2 | 55 | * |
|  |  | *Present* | 0.0 | 1 |  | 0.0 | 1 |  |
|  | *TEM-136* | *Absent* | 18.2 | 55 | * | 18.2 | 55 | * |
|  |  | *Present* | 0.0 | 1 |  | 0.0 | 1 |  |
|  | *TEM-157* | *Absent* | 20.0 | 5 | * | 20.0 | 5 | * |
|  |  | *Present* | 17.6 | 51 |  | 17.6 | 51 |  |
|  | *TEM-163* | *Absent* | 18.2 | 55 | * | 18.2 | 55 | * |
|  |  | *Present* | 0.0 | 1 |  | 0.0 | 1 |  |
|  | *bl2b* | *Absent* | 16.7 | 6 | * | 16.7 | 6 | * |
|  |  | *Present* | 18.0 | 50 |  | 18.0 | 50 |  |
| **p > 0.20*  ***p 0.20-0.10*  ****p < 0 .10* | . | | | | | | | |

N/A There was not observation for this combination of categories
